# Formative Research for the Development of the CHoBI7 Cholera Rapid Response Program for Cholera Hotspots in Bangladesh

**DOI:** 10.3390/ijerph192013352

**Published:** 2022-10-16

**Authors:** Fatema Zohura, Elizabeth D. Thomas, Jahed Masud, Md Sazzadul Islam Bhuyian, Tahmina Parvin, Shirajum Monira, Abu S. G. Faruque, Munirul Alam, Christine Marie George

**Affiliations:** 1 Research, Training and Management International, Dhaka 1216, Bangladesh; 2 International Centre for Diarrhoeal Disease Research, icddr,b, Dhaka 1212, Bangladesh; 3 Department of International Health, Johns Hopkins Bloomberg School of Public Health, Baltimore, MD 21205, USA

**Keywords:** mHealth, behavior change, cholera and diarrhea, handwashing with soap, water treatment, WASH, Bangladesh

## Abstract

Cholera is a severe form of acute watery diarrhea that if left untreated can result in death. Globally, there are 2.9 million cholera cases annually. Individuals living in close proximity to cholera cases are at a higher risk for developing cholera compared to the general population. Targeted water, sanitation, and hygiene (WASH) interventions have the potential to reduce cholera transmission in cholera hotspots around cholera cases. The objective of this study was to expand the scope of the Cholera-Hospital-Based-Intervention-for-7-Days (CHoBI7) program, focused on cholera patient households, for delivery in cholera hotspots in urban slums in Dhaka, Bangladesh. Thirty-one semi-structured interviews were conducted in cholera hotspots around cholera patients, and three intervention planning workshops were conducted to inform modifications needed to the CHoBI7 program. After exploratory interviews, a two-phase, iterative pilot study was conducted for 9 months to test the developed CHoBI7 Cholera Rapid Response program among 180 participants to further inform modifications to intervention content and delivery. Findings from pilot participant interviews highlighted the need to adapt intervention content for delivery at the compound—rather than household—level, given an environment with multiple households sharing a water source, toilets, and kitchen facilities. This was addressed by conducting a “ring session” for intervention delivery in cholera hotspots for households to discuss how to improve their shared facilities together and encourage a compound-level commitment to promoted WASH behaviors and placement of soapy water bottles in shared spaces. Based on the low number of soapy water bottles observed in communal spaces during the first iteration of the pilot, we also added context-specific examples using the narratives of families in mobile messages to encourage WASH behavioral recommendations. Formative research identified important considerations for the modifications needed to tailor the CHoBI7 program for delivery in cholera hotspots in urban Bangladesh.

## 1. Introduction

Cholera is a severe form of acute watery diarrhea; if left untreated it can result in death [1]. Globally, 2.9 million cholera cases and 95,000 cholera deaths occur annually in cholera-endemic countries [2]. In Bangladesh alone, there are estimated to be over 160,000 cholera cases and 5000 deaths each year [2]. Furthermore, more than 84 million people in Bangladesh live in districts classified as high risk for cholera (annual incidence rate > 3 per 1000) [3]. Risk factors for cholera include crowded housing and poor water quality, sanitation infrastructure, and hygiene practices [4,5,6,7,8,9,10,11]. Effective and targeted water, sanitation, and hygiene (WASH) interventions are needed to reduce the burden of cholera globally. Individuals living in close proximity to cholera cases are at a higher risk for developing cholera compared to the general population [12,13,14,15]. Studies in Bangladesh, India, Nepal, Chad, Haiti, and the Democratic Republic of the Congo indicate that the highest risk period of disease transmission is the month after cholera cases are first identified at health facilities [16,17,18,19]. In our recent evaluation of hospitalized cholera patients in Dhaka, Bangladesh, we found that 40% of cholera patients had at least one household member that also developed a cholera infection during the one week after the patient was admitted to the hospital [20]. Other studies have found that neighbors of cholera patients are also at high risk of developing cholera [2,19,21]. Areas close to cholera patients are often called “cholera hotspots” to indicate this higher cholera risk and case count. In urban slums of India individuals within 20 meters of a cholera patient household were found to be at 16 times higher risk of cholera than the general population during the month after the cholera patient was admitted to a health facility [16,19]. Despite the high risk of cholera transmission within hotspots, there have been few studies conducted to develop interventions to reduce transmission within this high-risk population, with most studies focusing solely on index case households [22].

### Cholera-Hospital-Based-Intervention-for-7-Days

In an effort to reduce cholera among the household members of cholera patients, our research team developed the Cholera-Hospital-Based-Intervention-for-7-Days (CHoBI7). CHoBI7 promotes handwashing with soap and drinking water treatment to cholera patient households during the 7-day high-risk period for cholera transmission after a laboratory-confirmed cholera patient has been admitted to a health facility. In a randomized controlled trial (RCT) of the intervention, the CHoBI7 program was found to significantly reduce symptomatic and overall cholera infections among household members of cholera patients [23]. CHoBI7 was then adapted for household members of diarrhea patients of all etiologies [24]. A mobile health (mHealth) component was also added to the program, to remove the need for home visits for program delivery, where enrolled households received WASH-related voice and text messages weekly for 12 months [23,25]. In the RCT of the CHoBI7 mHealth program, the intervention was found to significantly reduce diarrhea and childhood stunting [23].

In this present study, our objective was to expand the scope of the CHoBI7 program to those living in cholera hotspots around cholera patients to reduce cholera transmission in this high-risk setting. Here, we report on the formative research conducted to modify the CHoBI7 program for delivery in cholera hotspots in Dhaka, Bangladesh.

## 2. Methods

### 2.1. Study Overview

There were three components to this formative research: (1) exploratory research to inform modifications needed to deliver the CHoBI7 program in cholera hotspots; (2) intervention modification; and (3) a two-phase pilot study to test and refine the intervention (Table 1). We present methods for the exploratory component, then the process of intervention modification, and finally the methods for the two-phase iterative pilot study.

### 2.2. Ethical Approval

This study was approved by the Ethical Review Committees (ERC) of icddr,b (International Centre for Diarrhoeal Disease Research, Bangladesh) (Protocol PR-19105) and the Johns Hopkins Bloomberg School of Public Health (Protocol 9255). All study participants provided written informed consent or assent.

### 2.3. Component I: Exploratory Research

From December 2019 to January 2020, we conducted 8 semi-structured interviews with purposive sampling of caregivers of children under five years (6 females and 2 males) from households residing in cholera hotspots in slum areas in Dhaka, Bangladesh. We considered a cholera hotspot as a ring of 20 m around a laboratory confirmed cholera patient household. This ring size of 20 m was selected based on a previous study in urban slums of India, which found that this population was at high risk of cholera during the 7-day high-risk period after the index cholera patient in the ring was identified [16]. Cholera patients were identified through the icddr,b diarrhea surveillance system, where a stool sample is collected from every 50th patient and analyzed for a panel of enteric pathogens by bacterial culture and microscopy. *Vibrio cholerae* is one of the enteric pathogens assessed by bacterial culture through this surveillance system. To be eligible for interviews, participants had to: (1) not have a tap and basin with running water inside their home (mostly slum areas of Dhaka); (2) have at least one household member with ownership of an active mobile phone in their possession on the day of enrollment; (3) have a child under five years of age in their household (to tailor the intervention to caregivers of young children as done in previous CHoBI7 programs [20]); and (4) reside within 20 m of a cholera patient (reside in a cholera hotspot).

All interviews took place at a time convenient for participants at their place of residence. Interviews were conducted by research staff trained in qualitative data collection (two female research investigators and one male research officer). Key topics of the interview guide included cholera awareness, causes and prevention of cholera, water treatment, and handwashing practices. All interviews were conducted in Bangla and audio recorded. Audio recordings were then transcribed verbatim in Bangla and translated into English for analysis by study investigators.

Interviewers reviewed transcriptions and field notes line-by-line and summarized and organized findings in a matrix according to the levels and dimensions of the Integrated Behavioral Model for Water, Sanitation, and Hygiene (IBM-WASH) to facilitate interpretation of findings and identify intervention components that would require modification [26]. Study team members then reviewed the summary matrix and transcriptions and translations for completeness and reviewed data for additional themes.

### 2.4. Component II: CHoBI7 Program Modification for Cholera Hotspots

#### 2.4.1. Starting Point for the CHoBI7 Cholera Rapid Response Program

The starting point for the CHoBI7 Cholera Rapid Response program was the original CHoBI7 program for cholera patients and their household members [20], as well as the recent CHoBI7 mHealth program for diarrhea patients (of any etiology) and their household members [23,24,25]. A complete description of the interventions delivered in previous CHoBI7 programs is published elsewhere [20,25]. An overview of the main components is provided in Table 2.

#### 2.4.2. Behavioral Recommendations

Following previous CHoBI7 programs, the CHoBI7 Cholera Rapid Response program targets the following behaviors in cholera hotspots during the one month high-risk period after a cholera patient is identified: (1) preparing soapy water for handwashing using water and detergent powder [27]; (2) handwashing with soap at food- and stool-related events; (3) treating household drinking water using chlorine tablets; (4) safe drinking water storage in a water vessel with a lid and tap; and (5) boiling household drinking water until it reaches a rolling boil (large bubbles form) after the one month high-risk period.

#### 2.4.3. Intervention Components

Following the previous CHoBI7 programs, the CHoBI7 Cholera Rapid Response program was initially designed to include: (1) a pictorial module (flipbook); (2) an mHealth module (text and voice messages); (3) cue to action cards; and (4) a cholera prevention package to support adoption of recommended behaviors. The flipbook contains information on how severe diarrhea, including cholera, is transmitted in the environment, home, and compound, and how to prevent transmission. The mHealth module includes weekly text, voice, and interactive voice response (IVR) messages from three characters, Dr. Chobi, Aklima, and Aklima’s husband. Dr. Chobi is a doctor at icddr,b hospital who calls the participants to share information, encouragement, and reminders about the prevention of diarrhea. Aklima is a mother of a child hospitalized at icddr,b, who shares how she follows Dr. Chobi’s advice to successfully keep her family healthy after hospitalization [24,25]. Aklima’s husband serves as a male role model who practices the WASH behaviors recommended by Dr. Chobi to keep his family healthy and happy. For IVR messages, participants are asked questions about their knowledge and practice of recommended WASH behaviors. The cue to action cards and sticker provide visual instructions and serve as reminders to follow recommended handwashing and water treatment behaviors. The cholera prevention package includes a locally made handwashing station (16-L red plastic bucket with fixed tap and lid, basin, and stool to keep the bucket elevated), 500 mL soapy water bottle (25 gm detergent powder in 500 mL water), drinking water storage container (12-L blue plastic bucket with lid, fixed tap, and stool), chlorine tablets for treating drinking water, and a program sticker to encourage WASH behavioral recommendations.

#### 2.4.4. CHoBI7 Program Modification

Three intervention planning workshops, each lasting one day, were conducted among the research team to modify CHoBI7 intervention content based on exploratory research findings. During intervention planning workshops, modifications were made to the contents of the flipbook, mobile messages, cue to action cards, and the program sticker, guided by IBM-WASH.

### 2.5. Component III: CHoBI7 Cholera Rapid Response Program Pilot Study

#### 2.5.1. Phase 1 Pilot Study Design and Data Collection

From February to December 2020, trained research assistants recruited 171 participants from 67 households in 14 cholera hotspots in Dhaka slum areas for a pilot study of the CHoBI7 Cholera Rapid Response program. There were 4–5 households per cholera hotspot, depending on the number of households meeting our eligibility criteria (<20 m from a cholera patient household). For each hotspot, there was one standard message (i.e., standard of care) household receiving only the Bangladesh government’s standard recommendation on the use of oral rehydration solution (ORS) for rehydration in the case of diarrhea. Pilot study household eligibility criteria was the same as it was for exploratory interviews.

To learn about study participants’ experiences during the Phase 1 pilot study, we conducted 16 semi-structured follow-up interviews with participants (15 females and 1 male). The median time between enrollment and the follow-up interview was 35 days; the range was 29 days to 4 months. All interviews took place in-person in the participant’s household, following the same data collection methods used for exploratory interviews.

To assess the uptake of recommended handwashing with soap and water treatment behaviors, research staff conducted unannounced spot checks and 5 h structured observation at food- and stool-related events in all pilot households at 1 week, and 1 and 3 months after enrollment. Unannounced spot checks were always conducted before structured observation. Descriptive statistics were used to describe the frequency of the presence of soap in the kitchen and latrine areas of households and the presence of chlorine in stored household drinking water during spot checks and the frequency of participant handwashing with soap during structured observation. All Phase 1 pilot households were visited for spot checks and structured observations; however, some households and participants were not present during household visits. During the pilot, team members met weekly for debriefing to discuss intervention findings and further modifications needed for components of program delivery. Data collection activities were paused from March to May 2020 and June to September 2020 due to COVID-19 restrictions. 

#### 2.5.2. Phase 1 Pilot Study Intervention Delivery

The intervention was delivered across two household visits during the first 7 days of the high-risk period for cholera transmission: one visit within 24 h of enrollment and one follow-up visit 4–5 days after enrollment. During the first visit, a health promoter delivered the flipbook, introduced household members to the mHealth module characters and provided a training on how to receive calls, respond to IVR messages, and open text messages, and provided households with the cholera prevention package. One sticker and four cue cards were provided, showing pictures of: (1) the key times for handwashing with soap, (2) the importance of handwashing with soap during food preparation, (3) how to use chlorine tablets to treat drinking water, and (4) Dr. Chobi with the schedule for mobile message delivery. Promoters asked households to hang cue cards on the wall near the handwashing station and safe water storage container. During the second visit, promoters asked household members whether they had faced challenges with the hardware or recommendations, and problem-solved if any challenges were encountered.

#### 2.5.3. Phase 2 Pilot Study Design and Data Collection

From January to March 2021, because of COVID-19 lockdowns, we were only able to recruit 9 participants in 4 households from one cholera hotspot in a slum area in Dhaka, Bangladesh. These participants were enrolled following the same enrollment criteria as in the Phase 1 pilot study. In the Phase 2 pilot study, there were no standard message households. To learn about study participants’ experiences during the Phase 2 pilot study, 7 follow-up interviews were conducted with 5 participants (median 37.5 days between enrollment and follow-up interview; range 37 days to 4 months). Two study participants were interviewed twice because we wanted to obtain additional feedback on mobile message delivery. Two semi-structured interviews took place over the phone due to COVID-19 restrictions. Due to the COVID-19 lockdown during Phase 2 of the pilot study, we did not conduct a quantitative assessment.

## 3. Results

### 3.1. Exploratory Interviews Informing Intervention Development

#### 3.1.1. Awareness and Perceptions of Cholera and Severe Diarrhea

All participants interviewed had heard of cholera. Some participants had direct experience with the disease, while several had heard about it from others, such as neighbors, or from icddr,b (commonly referred to as the “Cholera Hospital”). One participant said that cholera was “an ancient disease”, though most spoke of cholera as a present-day disease. Most participants considered cholera to be a severe form of diarrhea, called “*patla paykhana*” (watery stool) in Bangla. Some participants said cholera was severe diarrhea accompanied by vomiting, and a few also mentioned severe dehydration as a symptom. In most interviews, “cholera” and “severe diarrhea” were referred to interchangeably. Participants thought that people of all ages were susceptible to cholera but saw children as the most likely to get cholera or severe diarrhea.

Cholera was generally seen as a dangerous disease requiring treatment or hospitalization. While discussing their own experiences with cholera and severe diarrhea, two participants mentioned recent deaths from severe diarrhea in their neighborhoods. Cholera was considered treatable by intravenous and/or oral saline (ORS). Participants were familiar with the icddr,b Dhaka hospital and some had sought treatment for family members there in the past. Some participants mentioned getting oral saline from a pharmacy first, and then going to a hospital if the diarrhea did not stop.

When asked what they would do if someone got cholera in their neighborhood, most participants said that they would encourage the ill person to seek treatment at icddr,b. Most participants were aware that cholera could spread easily in neighborhoods, and three participants said they would need to “stay away” from people with cholera.

#### 3.1.2. Causes and Prevention of Cholera

When asked about causes of cholera, participants mentioned eating leftover or rotten food, street food, or food from shops. A few participants said that living in unhygienic conditions could cause cholera, such as living in a place with garbage, flies, and mosquitoes. Food hygiene, hand hygiene, and unsafe drinking water were also mentioned as causes of cholera or severe diarrhea. Similarly, participants said that maintaining cleanliness in living areas, washing hands with soap after toileting and before eating, drinking boiled water, and not eating stale or rotten food were important for diarrhea prevention.

#### 3.1.3. Handwashing with Soap and Water Treatment

One participant noted that handwashing facilities near toilets were inconvenient and crowded, which meant a long wait to wash hands after toileting. Participants noted other barriers to handwashing with soap, including lack of time, “laziness”, and the cost of soap.

*“Many people do not do [wash hands] …sometimes, I also do not wash hands [with soap]. (Participant laughs) Yes, it happens sometimes. I myself also make mistakes. …Sometimes it happens that I did not wash my hands after coming from the toilet, as I was in a hurry, my child was crying and I needed to stop her…Yes, I couldn’t [wash my hands] due to lack of time. (Participant laughs) …No other reasons, only lack of time.” Female, cholera hotspot household, Age 30*

One participant said that others would be encouraged and learn how to wash their hands if they saw more people doing it. Participants said they received information on washing hands from the hospital and from Lifebuoy or Dettol liquid soap advertisements on the television. 

Drinking water available through the Dhaka municipal city was generally seen as unsafe, not well-maintained, and “dirty”. Boiling or filtering drinking water were water treatment practices reported among participants, though one cited the availability of gas as a barrier to boiling water.

### 3.2. CHoBI7 Program Modification for Cholera Hotspots

#### 3.2.1. Information on Cholera and Cholera Prevention

To adapt intervention content for households living in cholera hotspots, behavioral recommendations were framed around the high risk of cholera over the next month due to a cholera patient residing in the neighborhood. Based on the findings from the exploratory research that cholera was often referred to as severe diarrhea, we mentioned both cholera and severe diarrhea in all behavior change communication. In the flipbook and mHealth module, we provided information about mortality risk among all age groups due to cholera to increase awareness of the severity of the disease. To adapt the mHealth messages for cholera hotspots, we changed the content to focus on households “being in a high-risk area for cholera due to a cholera case being found in close proximity to their household” rather than “living in a household with a cholera patient.” 

#### 3.2.2. The Story of the Busy Family

During exploratory interviews, participants mentioned that they were “too busy” to wash their hands with soap or boil water for drinking. Lack of time is a commonly cited barrier to performing WASH behaviors [24,28]. Given this, we added a story to the flipbook about a busy family with two young children who were living in a cholera hotspot in Dhaka, Bangladesh. In this story, the CHoBI7 intervention team visited their household and told them they were at high risk for cholera because there was a recent cholera patient in their area. The household members replied that they were too busy to worry about handwashing with soap and water treatment. A few days later, both children fell ill with severe diarrhea and had to be rushed to the hospital. The hospital said they had cholera. The mother also became ill with cholera while in the hospital caring for her children. The father could not go to work because he had to take care of his family, which added economic strain to the family. The story serves as a cautionary tale for those saying that they lack time for washing hands with soap and treating drinking water.

### 3.3. Pilot Phase 1 Findings

#### 3.3.1. Uptake of Behavioral Recommendations and Use of Cholera Prevention Package

At the Day 7 follow-up visit after enrollment of cholera hotspot households, we observed that 76% (67/88) of study participants in the intervention arm washed their hands with soap at key food- and stool-related events compared to 57% (16/28) in the standard message arm. At Month 1, we observed that 67% (56/83) of study participants in the intervention arm washed their hands with soap at key food- and stool-related events compared to 33% (9/24) in the standard message arm. Finally, at Month 3, we observed that 61% (39/64) of study participants in the intervention arm washed their hands with soap at key food- and stool-related events compared to 41% (9/22) in the standard message arm.

At Day 7 in intervention households, 98% (55/56) had a soapy water bottle present in their household. At Month 1 and Month 3, this was 96% (46/48) and 89% (35/39), respectively. No standard arm households had a soapy water bottle present. In the communal kitchen and toilet areas of study compounds, soapy water bottle presence was lower (Day 7: kitchen area 67% (37/56), bathroom area 55% (31/56); Month 1: kitchen area 71% (38/48), bathroom area 54% (26/48); Month 3: kitchen area 77% (30/39), bathroom area 64% (25/39)).

For water treatment, 79% (44/56) of stored water samples collected from intervention households at the 7-day follow-up had detectable free available chlorine. This dropped to 23% (11/48) of samples at Month 1 and 0% (0/39) at Month 3 (in line with when the chlorine tablets provided for a 7-day period would have been all used).

#### 3.3.2. Participants’ Experiences, Preferences, and Recommendations for Intervention Delivery

##### Handwashing with Soap

Pilot participants reported barriers to handwashing with soap, including the absence of soap in or near to the communal toilet and long queues to wash hands at communal toilets.

*“It would not be difficult [to wash hands], but people could think that ‘I am busy now and I have to wait 5 min for washing hands at the [shared] toilet.’” Female, cholera hotspot household, Age 25*

Participants also mentioned the ease of having a handwashing station in their home.

*“This [handwashing station] is very good... Look, we don’t have personal toilets, we have the shared toilet facility [in the compound]. Sometimes it is very difficult to wash hands if it is locked. Now, it is very helpful for us. No matter whether people are in the toilet or not, we can wash our hands by using this water [handwashing station in the home], which is very helpful. Because, it is not our personal toilet, we are three families using this toilet.” Female, cholera hotspot household, Age 36*

Pilot participants mentioned that their handwashing station facilitated handwashing with soap for both adults and children and served as a reminder to wash hands with soap. Two pilot participants thought that people were more aware of handwashing with soap due to the ongoing COVID-19 pandemic.

##### Soapy Water Bottle

The soapy water bottle was viewed as more convenient and less expensive than bar soap. Pilot participants said they could easily prepare the soapy water and keep the bottles in the toilet, inside their rooms, and the kitchen areas for washing their hands.

##### Water Treatment

Most pilot participants said they had already been treating their drinking water by boiling or filtering before receiving the chlorine tablets as part of the intervention. A few pilot participants said they were previously drinking direct water from the tap (*koler pani* in Bangla) or tube wells. Two participants who said they were using the chlorine tablets from the CHoBI7 program expressed concern about their availability in local markets. Another participant mentioned that chlorine tablets could be a safe alternative to boiling water, which could result in burning accidents. Availability of gas was also mentioned by pilot study participants as a barrier to boiling drinking water.

##### Cue Cards

One participant suggested adding a picture of germs to the cue cards to highlight the importance of handwashing. 

### 3.4. Further Program Modification

Based on the findings from the Phase 1 pilot, we further revised CHoBI7 intervention content and program delivery.

#### 3.4.1. Ring Session

Based on the Phase 1 pilot study findings about long queues for handwashing at shared facilities, and the low proportion of hotspots with soapy water bottles in communal spaces, we introduced a group session for intervention delivery in cholera hotspots to give households an opportunity to discuss how to improve their shared facilities together and encourage a compound-level commitment to perform the promoted WASH behaviors. We called this a “ring session”, as we recruited neighbors from a 20 m ring around a cholera patient. The ring session took place on Day 2 of the intervention, after the first household visit; all enrolled household members were invited. During the ring session, the health promoter delivered a short flipbook module on cholera transmission and why participants are at a high risk of cholera. A demonstration session was conducted on how to wash hands with soap/soapy water, treat water using chlorine tablets, and prepare soapy water, where session participants showed others in the session how to correctly perform these behaviors. Participants were encouraged to keep soapy water bottles in communal spaces. A mobile message from Dr. Chobi was played during the session and discussed. Health promoters also facilitated discussion among participants on any intervention challenges encountered since intervention delivery.

#### 3.4.2. The Story of The Hygiene Champion

In the ring session, “The Story of the Hygiene Champion” was included as a narrative to facilitate a discussion around handwashing with soap and water treatment in shared facilities. The vignette features the mHealth character Aklima, and tells a story of how her neighbor, Shahnaz, and her family became ill with cholera after not practicing handwashing with soap and water treatment. However, after following the guidance of Dr. Chobi, the family’s children stayed healthy and exceled in school.

#### 3.4.3. Hanging Multiple Soapy Water Bottles

Given the low rates of soapy water bottles in communal cooking and toileting areas during Phase 1 pilot study spot checks, we added a specific recommendation to the flipbook and mHealth messages about preparing and hanging multiple soapy water bottles on a string in those locations. The recommendation to hang soapy water bottles with string was based on previous study findings that young children play with soapy water bottles and spill the water.

#### 3.4.4. New Cue Card

In an effort to increase handwashing with soap at food- and stool-related events from the rates found in our quantitative assessment in Phase 1 of the pilot study, and based on the suggestion from a pilot participant, we also developed a new cue card showing a hand with visible germs, and explained that germs are present on our hands even when they are not visible to the naked eye.

#### 3.4.5. Emphasis on COVID-19

To leverage ongoing WASH programs promoting handwashing with soap for COVID-19 prevention, we added to the flipbook and mHealth modules that handwashing with soap was important not only for cholera and severe diarrhea but also for COVID-19 prevention.

### 3.5. Pilot Phase 2 Findings

In the Phase 2 pilot study, the ring session was well received; pilot participants felt they benefited from engagement with other households in the intervention.

*“It feels good that we gathered in one place [the ring session]...we had a discussion together and got advice. We were able to learn good and bad things about cholera disease. …I liked that moment [the ring session] where we all [neighbors] talked together.” Female, cholera hotspot household, Age 19*

Participants said that having multiple soapy water bottles on strings in communal places near latrines and cooking areas was beneficial. The story of the hygiene champion told during the ring session was also well received.

*“I have learned from the story of the champion [about Aklima’s neighbor] that we have to be careful about them [neighbors] …who are not following the instructions of Dr. Chobi Apa.” Female, cholera hotspot household, Age 19*

There were no recommendations made by participants on modifications to the intervention. Therefore, no additional changes were made to intervention materials based on Phase 2 piloting.

## 4. Discussion

Formative research was critical for tailoring the CHoBI7 program for those residing in cholera hotspots around cholera patients. Exploratory interviews provided valuable insights on cholera awareness in the community, and the challenges of practicing WASH behaviors in an environment where both latrine and kitchen areas are communal. Through our two-phase pilot study, we were able to make adaptions to intervention components based on participant feedback and household assessments. The ring session was a valuable addition to program delivery that allowed for engagement of households residing in the same cholera hotspot. In addition, including narratives of the stories of households residing in cholera hotspots in mobile messages was considered a valuable addition by pilot participants. The findings from this study demonstrate that the CHoBI7 Cholera Rapid Response program was feasible and acceptable for a population at high-risk for cholera in slum areas of Dhaka, Bangladesh. These findings complement our recent RCT of the CHoBI7 Cholera Rapid Response program, which showed that this program was effective in significantly reducing diarrhea and increasing handwashing with soap and water treatment practices in cholera hotspots in Bangladesh [29].

The findings from this study build on our previous CHoBI7 formative work, which focused on diarrhea patient households [24,25]. Many of the facilitators and barriers to the promoted WASH behaviors found in our previous work were similar for those living in cholera hotspots. This is likely because participants of both studies share a similar household environment in slum areas of Dhaka. We are not aware of another study that conducted formative research to develop a WASH program for those living in cholera hotspots. Formative research is needed to design evidence-based intervention approaches to support adoption of preventative WASH behaviors in cholera hotspots globally.

A crucial factor determining the success of WASH programs targeting cholera hotspots is early detection and rapid response [22]. In Haiti, prompt delivery of WASH rapid response teams reduced cholera outbreak duration in cholera hotspots [30]. However, in Nepal and Yemen, challenges with coordination between relevant actors when a cholera case was identified delayed WASH program delivery in cholera hotspots, hampering successful program implementation [22]. In our present study, the CHoBI7 Cholera Rapid Response program was delivered to households in cholera hotspots within 48–72 h of admission of a cholera patient to a health facility—allowing for rapid program delivery. Future research is needed to determine approaches to rapidly deliver WASH programs in cholera hotspots globally.

In the CHoBI7 Cholera Rapid Response program, we added context-specific examples using the narratives of families to reinforce CHoBI7 behavioral recommendations on handwashing with soap, water treatment, and safe water storage in mobile messages. Previous studies have found narratives to be a valuable tool for health promotion in the public health field by motivating and supporting behavior change [31,32,33,34,35,36]. Narratives have frequently been used for smoking cessation programs [30,31,33], sexual health promotion [37,38], and cancer prevention and control [39,40]. Future studies could compare whether the inclusion of narratives facilitates WASH behavior change in cholera hotspots by comparing intervention delivery with and without this component.

“*Ending Cholera—A Global Roadmap to 2030*”, developed by the Global Task Force on Cholera Control (GTFCC), is a global strategy for cholera control [41]. The goal is to reduce cholera deaths by 90% and have 20 countries eliminate cholera by 2030. The roadmap recommends that countries focus on reducing cholera in cholera hotspots, given that these high-risk areas can spread cholera to other regions, and recommends that cholera rapid response teams deliver WASH and oral cholera vaccine in cholera hotspots. Therefore, the findings from this study are of public health importance globally, given the need to develop evidence-based approaches to reduce cholera in hotspots worldwide.

This study has some strengths. First, the iterative nature of intervention development based on exploratory research and two phases of intervention piloting helped us to identify the target community’s perspective on cholera and recommendations on intervention approaches in cholera hotspots. Second, the use of structured observation and unannounced spot checks to determine intervention uptake, and interviews to explore participants’ experiences with the recommended WASH behaviors, allowed for a mixed methods approach for evaluating the pilot of the CHoBI7 Cholera Rapid Response program. Finally, implementing an intervention approach at both the household and compound levels allowed for a multi-level approach for program implementation, building on our previous CHoBI7 work focused on diarrhea patient households.

This study also has some limitations. First, we conducted follow-up interviews with mostly adult women (28 females and 03 males) due to the unavailability of male participants. Future studies should try to include more males living in cholera hotspots to ensure interventions are tailored to them as well, and to better understand their facilitators and barriers to performing the recommended WASH behaviors in the household and shared facilities. Second, lockdowns due to the COVID-19 pandemic limited the Phase 2 pilot study to one cholera hotspot. These restrictions also limited our ability to fully explore participants’ responses to modified intervention materials, including narratives. Future research could be done with participants of the recent RCT of the CHoBI7 Cholera Rapid Response Program to better understand responses to all intervention components. Third, a limitation of structured observations is the Hawthorne effect. However, structured observation is considered to be the gold standard for collecting handwashing with soap data, and the alternative of self-reported handwashing practices is prone to social desirability bias.

## 5. Conclusions

Formative research identified important considerations for the modifications that needed to be made to tailor the CHoBI7 program for delivery in cholera hotspots in Bangladesh. We observed high acceptability of the CHoBI7 Cholera Rapid Response Program among participants, and found this program was feasible to implement in our study setting. This study provides a model that can be used for the development of WASH interventions for other cholera hotspots globally.

## Figures and Tables

**Table 1:** Semi-structured interviews conducted with community members residing in cholera hotspots.

**Exploratory Interviews (December 2019 to January 2020)** Explore cholera awareness, causes and prevention of cholera, and existing water treatment and handwashing practices	**Female Household Members**	**Male Household Members**	**Total**
	6	2	8
**Pilot Interviews (February to December 2020)** Explore participants’ experiences, including feasibility and acceptability, with the CHoBI7 Cholera Rapid Response program	**Female Household Members**	**Male Household Members**	**Total**
		
**Pilot Phase 1**	15	1	16
**Pilot Phase 2**	7	0	7

**Table 2:** Overview of CHoBI7 program activities.

	Original CHoBI7 Program		CHoBI7 mHealth Program			CHoBI7 Cholera Rapid Response Program	
	Standard Arm	Intervention Arm	Standard Arm	mHealth with No Home Visits Arm	mHealth with Two Home Visits Arm	Standard Arm	Intervention Arm
**Key Components**	1 health facility-based visit	1 health facility-based visit + 3 home visits	1 health facility-based visit	1 health facility-based visit + mHealth program	1 health facility-based visit + 2 home visits + mHealth program	1 home visit	2 home visits + 1 ring session + mHealth program
**Intervention Activities**	▪ Deliver the standard message on use of oral rehydration solution (ORS) for rehydration	▪ Deliver the standard message on use of ORS for dehydration ▪ Provide CHoBI7 Cholera Prevention Package ▪ Deliver CHoBI7 Health Facility Flipbook module ▪ Deliver CHoBI7 Household Flipbook module	▪ Deliver the standard message on use of ORS for rehydration	▪ Deliver the standard message on use of ORS for rehydration ▪ Provide CHoBI7 Diarrhea Prevention Package ▪ Deliver CHoBI7 Health Facility Flipbook module ▪ Deliver CHoBI7 mHealth module (weekly phone calls, text messages, and interactive voice response (IVR) quizzes) for 12 months	▪ Deliver the standard message on use of ORS for rehydration ▪ Provide CHoBI7 Diarrhea Prevention Package ▪ Deliver CHoBI7 Health Facility Flipbook module ▪ Deliver CHoBI7 Household Flipbook module ▪ Deliver CHoBI7 mHealth module (weekly phone calls, text messages, and IVR quizzes) for 12 months	▪ Deliver the standard message on use of ORS for rehydration	▪ Deliver the standard message on use of ORS for rehydration ▪ Provide CHoBI7 Cholera Prevention Package ▪ Conduct ring session with demonstration of key WASH behaviors ▪ Deliver CHoBI7 Household Flipbook module ▪ Deliver CHoBI7 mHealth module (weekly phone calls, text messages, and IVR quizzes) for 3 months

## Data Availability

The data supporting the conclusions of this article are available from the corresponding author upon reasonable request.

## References

[1] Sack D.A., Sack R.B., Nair G.B., Siddique A.K. (2004). Cholera. Lancet.

[2] Ali M., Nelson A.R., Lopez A.L., Sack D.A. (2015). Updated Global Burden of Cholera in Endemic Countries. PLoS Negl. Trop. Dis..

[3] International Vaccine Institute (2013). Country Investment Case Study on Cholera Vaccination: Bangladesh.

[4] Blake P.A., Ramos S., MacDonald K.L., Rassi V., Gomes T.A.T., Ivey C., Bean N.H., Trabulsi L.R. (1993). Pathogen-specific risk factors and protective factors for acute diarrheal disease in urban Brazilian infants. J. Infect. Dis..

[5] Esrey S.A. (1996). Water, waste, and well-being. A multicountry study. Am. J. Epidemiol..

[6] D’souza R.M. (1997). Housing and environmental factors and their effects on the health of children in the slums of Karachi, Pakistan. J. Biosoc. Sci..

[7] Saha D. (2013). Acute Diarrhoea in Children in Rural Gambia: Knowledge, Attitude and Practice, Aetiology, Risk Factors and Consequences among Children Less than Five Years of Age.

[8] Tornheim J.A., Morland K.B., Landrigan P.J., Cifuentes E. (2009). Water privatization, water source, and pediatric diarrhea in Bolivia. Epidemiologic analysis of a social experiment. Int. J. Occup. Environ. Health.

[9] George C.M., Perin J., De Calani K.J.N., Norman W.R., Perry H., Davis T.P., Lindquist E.D. (2014). Risk factors for diarrhea in children under five years of age residing in peri-urban communities in Cochabamba, Bolivia. Am. J. Trop. Med. Hyg..

[10] Clasen T., Saeed T.F., Boisson S., Edmondson P., Shipin O. (2007). Household water treatment using sodium dichloroisocy anurate (NaDCC) tablets: A randomized, controlled trial to assess microbiological effectiveness in Bangladesh. Am. J. Trop. Med. Hyg..

[11] Sirajul Islam M., Brooks A., Kabir M., Jahid I., Shafiqul Islam M., Goswami D., Nair G., Larson C., Yukiko W., Luby S. (2007). Faecal contamination of drinking water sources of Dhaka city during the 2004 flood in Bangladesh and use of disinfectants for water treatment. J. Appl. Microbiol..

[12] Hughes J.M., Boyce J.M., Levine R.J., Khan M., Aziz K., Huq M., Curlin G.T. (1982). Epidemiology of eltor cholera in rural Bangladesh: Importance of surface water in transmission. Bull. World Health Organ..

[13] Weil A.A., Khan A.I., Chowdhury F., LaRocque R.C., Faruque A., Ryan E.T., Calderwood S.B., Qadri F., Harris J.B. (2009). Clinical outcomes in household contacts of patients with cholera in Bangladesh. Clin. Infect. Dis..

[14] Spira W.M., Khan M.U., Saeed Y.A., Sattar M.A. (1980). Microbiological surveillance of intra-neighbourhood E1 Tor cholera transmission in rural Bangladesh. Bull. World Health Organ..

[15] Glass R.I., Svennerholm A.-M., Khan M., Huda S., Imdadul Huq M., Holmgren J. (1985). Seroepidemiological studies of EI Tor cholera in Bangladesh: Association of serum antibody levels with protection. J. Infect. Dis..

[16] Ali M., Debes A.K., Luquero F.J., Kim D.R., Park J.Y., Digilio L., Manna B., Kanungo S., Dutta S., Sur D. (2016). Potential for controlling cholera using a ring vaccination strategy: Re-analysis of data from a cluster-randomized clinical trial. PLoS Med..

[17] Azman A.S., Luquero F.J., Salje H., Mbaïbardoum N.N., Adalbert N., Ali M., Bertuzzo E., Finger F., Toure B., Massing L.A. (2018). Micro-hotspots of risk in urban cholera epidemics. J. Infect. Dis..

[18] Finger F., Bertuzzo E., Luquero F.J., Naibei N., Touré B., Allan M., Porten K., Lessler J., Rinaldo A., Azman A.S. (2018). The potential impact of case-area targeted interventions in response to cholera outbreaks: A modeling study. PLoS Med..

[19] Debes A.K., Ali M., Azman A.S., Yunus M., Sack D.A. (2016). Cholera cases cluster in time and space in Matlab, Bangladesh: Implications for targeted preventive interventions. Int. J. Epidemiol..

[20] George C.M., Monira S., Sack D.A., Rashid M.U., Saif-Ur-Rahman K.M., Mahmud T., Rahman Z., Mustafiz M., Bhuyian S.I., Winch P.J. (2016). Randomized Controlled Trial of Hospital-Based Hygiene and Water Treatment Intervention (CHoBI7) to Reduce Cholera. Emerg. Infect. Dis..

[21] Roskosky M., Acharya B., Shakya G., Karki K., Sekine K., Bajracharya D., Von Seidlein L., Devaux I., Lopez A.L., Deen J. (2019). Feasibility of a comprehensive targeted cholera intervention in the Kathmandu Valley, Nepal. Am. J. Trop. Med. Hyg..

[22] Sikder M., Altare C., Doocy S., Trowbridge D., Kaur G., Kaushal N., Lyles E., Lantagne D., Azman A.S., Spiegel P. (2021). Case-area targeted preventive interventions to interrupt cholera transmission: Current implementation practices and lessons learned. PLoS Negl. Trop. Dis..

[23] George C.M., Monira S., Zohura F., Thomas E.D., Hasan M.T., Parvin T., Hasan K., Rashid M.U., Papri N., Islam A. (2020). Effects of a Water, Sanitation and Hygiene Mobile Health Program on Diarrhea and Child Growth in Bangladesh: A Cluster-Randomized Controlled Trial of the CHoBI7 Mobile Health Program. Clin. Infect. Dis..

[24] Thomas E.D., Zohura F., Hasan M.T., Rana M.S., Teman A., Parvin T., Masud J., Bhuyian M.S.I., Hossain M.K., Hasan M. (2020). Formative research to scale up a handwashing with soap and water treatment intervention for household members of diarrhea patients in health facilities in Dhaka, Bangladesh (CHoBI7 program). BMC Public Health.

[25] George C.M., Zohura F., Teman A., Thomas E., Hasan T., Rana S., Parvin T., Sack D.A., Bhuyian S.I., Labrique A. (2019). Formative research for the design of a scalable water, sanitation, and hygiene mobile health program: CHoBI7 mobile health program. BMC Public Health.

[26] Dreibelbis R., Winch P.J., Leontsini E., Hulland K.R., Ram P.K., Unicomb L., Luby S.P. (2013). The integrated behavioural model for water, sanitation, and hygiene: A systematic review of behavioural models and a framework for designing and evaluating behaviour change interventions in infrastructure-restricted settings. BMC Public Health.

[27] Amin N., Pickering A.J., Ram P.K., Unicomb L., Najnin N., Homaira N., Ashraf S., Abedin J., Islam M.S., Luby S.P. (2014). Microbiological evaluation of the efficacy of soapy water to clean hands: A randomized, non-inferiority field trial. Am. J. Trop. Med. Hyg..

[28] Parveen S., Nasreen S., Allen J.V., Kamm K.B., Khan S., Akter S., Lopa T.M., Zaman K., El Arifeen S., Luby S.P. (2018). Barriers to and motivators of handwashing behavior among mothers of neonates in rural Bangladesh. BMC Public Health.

[29] George C.M., Parvin T., Bhuyian M.S.I., Uddin I.M., Zohura F., Masud J., Monira S., Sack D.A., Perin J., Alam M. (2022). Randomized Controlled Trial of the Cholera-Hospital-Based-Intervention-for-7-Days (CHoBI7) Cholera Rapid Response Program to Reduce Diarrheal Diseases in Bangladesh. Int. J. Environ. Res. Public Health.

[30] Michel E., Gaudart J., Beaulieu S., Bulit G., Piarroux M., Boncy J., Dely P., Piarroux R., Rebaudet S. (2019). Estimating effectiveness of case-area targeted response interventions against cholera in Haiti. eLife.

[31] Kim H.S., Bigman C.A., Leader A.E., Lerman C., Cappella J.N. (2012). Narrative Health Communication and Behavior Change: The Influence of Exemplars in the News on Intention to Quit Smoking. J. Commun..

[32] Remein C.D., Childs E., Pasco J.C., Trinquart L., Flynn D.B., Wingerter S.L., Bhasin R.M., Demers L.B., Benjamin E.J. (2020). Content and outcomes of narrative medicine programmes: A systematic review of the literature through 2019. BMJ Open.

[33] Fu S.S., Rhodes K.L., Robert C., Widome R., Forster J.L., Joseph A.M. (2014). Designing and evaluating culturally specific smoking cessation interventions for American Indian communities. Nicotine Tob. Res..

[34] Laskow T., Small L., Wu D.S. (2019). Narrative Interventions in the Palliative Care Setting: A Scoping Review. J. Pain Symptom Manag..

[35] Zhou C., Occa A., Kim S., Morgan S. (2020). A Meta-analysis of Narrative Game-based Interventions for Promoting Healthy Behaviors. J. Health Commun..

[36] Hinyard L.J., Kreuter M.W. (2007). Using narrative communication as a tool for health behavior change: A conceptual, theoretical, and empirical overview. Health Educ. Behav..

[37] (1999). Community-level HIV intervention in 5 cities: Final outcome data from the CDC AIDS Community Demonstration Projects. Am. J. Public. Health.

[38] Mevissen F.E.F., Ruiter R.A.C., Meertens R.M., Schaalma H.P. (2010). The effects of scenario-based risk information on perceptions of susceptibility to Chlamydia and HIV. Psychol. Health.

[39] Sitto K., Lubinga E., Geya M. (2021). The power of narrative health communication: Exploring possible effects of first-hand experiential stories on cancer awareness amongst university students. TD J. Transdiscipl. Res. South. Afr..

[40] Kreuter M.W., Green M.C., Cappella J.N., Slater M.D., Wise M.E., Storey D., Clark E.M., O’Keefe D.J., Erwin D.O., Holmes K. (2007). Narrative communication in cancer prevention and control: A framework to guide research and application. Ann. Behav. Med..

[41] World Health Organization (2019). Global Task Force on Cholera Control. Ending Cholera: A Global Roadmap to 2030.

